# Exploration of Potential Roles of m5C-Related Regulators in Colon Adenocarcinoma Prognosis

**DOI:** 10.3389/fgene.2022.816173

**Published:** 2022-02-24

**Authors:** Yuancheng Huang, Chaoyuan Huang, Xiaotao Jiang, Yanhua Yan, Kunhai Zhuang, Fengbin Liu, Peiwu Li, Yi Wen

**Affiliations:** ^1^ First Clinical Medical College, Guangzhou University of Chinese Medicine, Guangzhou, China; ^2^ Department of Gastroenterology, Baiyun Branch of the First Affiliated Hospital of Guangzhou University of Chinese Medicine, Guangzhou, China; ^3^ Department of Gastroenterology, The First Affiliated Hospital of Guangzhou University of Chinese Medicine, Guangzhou, China

**Keywords:** colon adenocarcinoma, epigenetics, RNA modification, 5-methylcytosine, gene expression profile, prognostic signature, TCGA

## Abstract

**Objectives:** The purpose of this study was to investigate the role of 13 m^5^C-related regulators in colon adenocarcinoma (COAD) and determine their prognostic value.

**Methods:** Gene expression and clinicopathological data were obtained from The Cancer Genome Atlas (TCGA) datasets. The expression of m^5^C-related regulators was analyzed with clinicopathological characteristics and alterations within m^5^C-related regulators. Subsequently, different subtypes of patients with COAD were identified. Then, the prognostic value of m^5^C-related regulators in COAD was confirmed *via* univariate Cox regression and least absolute shrinkage and selection operator (LASSO) Cox regression analyses. The prognostic value of risk scores was evaluated using the Kaplan-Meier method, receiver operating characteristic (ROC) curve. The correlation between the two m^5^C-related regulators, risk score, and clinicopathological characteristics were explored. Additionally, Gene Set Enrichment Analysis (GSEA), Kyoto Encyclopedia of Genes and Genomes (KEGG) pathways, and Gene Ontology (GO) analysis were performed for biological functional analysis. Finally, the expression level of two m^5^C-related regulators in clinical samples and cell lines was detected by quantitative reverse transcription-polymerase chain reaction and through the Human Protein Atlas database.

**Results:** m^5^C-related regulators were found to be differentially expressed in COAD with different clinicopathological features. We observed a high alteration frequency in these genes, which were significantly correlated with their mRNA expression levels. Two clusters with different prognostic features were identified. Based on two independent prognostic m^5^C-related regulators (NSUN6 and ALYREF), a risk signature with good predictive significance was constructed. Univariate and multivariate Cox regression analyses suggested that the risk score was an independent prognostic factor. Furthermore, this risk signature could serve as a prognostic indicator for overall survival in subgroups of patients with different clinical characteristics. Biological processes and pathways associated with cancer, immune response, and RNA processing were identified.

**Conclusion:** We revealed the genetic signatures and prognostic values of m^5^C-related regulators in COAD. Together, this has improved our understanding of m^5^C RNA modification and provided novel insights to identify predictive biomarkers and develop molecular targeted therapy for COAD.

## Introduction

Changes in gene expression are closely associated with the development of disease, and epigenetic processes are heritable changes in gene expression that do not alter the nucleotide sequence (Wu and Morris, 2001). Traditional epigenetic modifications, including chromatin remodeling, DNA methylation, and histone modifications, are involved in various biological processes related to the occurrence and progression of tumors, including gastrointestinal cancers (Dawson and Kouzarides, 2012; Darwiche, 2020; Grady et al., 2021). With considerable progress in zymology and high-throughput sequencing technology, epitranscriptomics has attracted significant attention recently (Angelova et al., 2018; Porcellini et al., 2018; Minervini et al., 2020; Song et al., 2020; Zhao et al., 2020; Schaefer, 2021). Research investigating the physiological and pathological functions of RNA modifications have identified multiple dynamic modifications of RNA, including N6-methyladenosine, 2-O-dimethyladenosine, 5-methylcytosine (m^5^C), 7-methylguanosine, N1-methyladenosine, and pseudouridylation (Roundtree et al., 2017; Shi et al., 2020). Increasing evidence suggests that RNA modifications play critical roles in tumorigenesis and the progression of different cancers (Barbieri and Kouzarides, 2020; Begik et al., 2020). m^5^C RNA modification is found in a variety of RNAs, including messenger RNAs, transfer RNAs, ribosomal RNAs. This modification introduces a methyl group in the fifth carbon atom of cytosine (Yu-Sheng Chen et al., 2021). Based on published data, m^5^C RNA modification plays a critical role in the translation, transport, and stability of mRNAs, and is also closely associated with the biogenesis and function of other RNA species (Xue et al., 2020; Hussain, 2021). As a dynamic and reversible process, m^5^C RNA modification is primarily regulated by “writers” (adenosine methyltransferases) and “erasers” (demethylases), and achieves different functions by interacting with “readers” (m^5^C-binding proteins). The “writers” include the NOL1/NOP2/Sun domain RNA methyltransferase family NSUN1-NSUN7 and DNMT2. m^5^C “erasers” include enzymes in the TET family (TET1, TET2, TET3) and ALKBH1. The “readers”, such as ALYREF and YBX1, recognize and bind to methylated RNAs to realize different functions (Nombela et al., 2021; Xie et al., 2020).

Globally, colorectal cancer (CRC) is the third most common cancer and the second most deadly neoplasm (Bray et al., 2018). Colon adenocarcinoma (COAD) is the most common pathological type of CRC, and despite considerable progress in diagnosis and therapeutic strategies for COAD, the prognosis of patients with COAD remains poor due to advanced stage and postsurgical recurrence (White et al., 2017; Miller et al., 2019). Therefore, the identification of novel biomarkers for early detection and effective therapeutic targets for treating patients with COAD is critical and urgent.

In this study, we analyzed a TCGA dataset for m^5^C-related regulators involved in COAD, the correlation between the expression levels of 13 m^5^C-related regulators and clinicopathological features, as well as potential independent prognostic m^5^C-related regulators and a risk signature to predict the prognosis of patients with COAD.

## Material and Methods

### Acquisition of Datasets

The RNA-seq transcriptome data (fragments per kilobase million, FPKM) from 437 samples (Mortazavi et al., 2008), copy number variant (CNV) data from 825 samples, single nucleotide variant (SNV) data from 399 samples, and clinical information from 385 patients with COAD in TCGA database (http://cancergenome.nih.gov/) were downloaded for our study. Patients with complete clinicopathological and survival information were included for further assessment (Table 1).

**TABLE 1:** Clinicopathological features of patients included in this study.

	Total patients (337)		High-risk group (163)		Low-risk group (168)		*p*-value
	Number	Percentage (%)	Number	Percentage (%)	Number	Percentage (%)	
Fustat							0.009
Alive	279	82.8	125	76.7	148	88.1	
Dead	58	17.2	38	23.3	20	11.9	
Age							0.178
≤65	135	40.1	72	44.2	61	36.3	
>65	202	59.9	91	55.8	107	63.7	
gender							0.714
female	156	46.3	78	47.9	76	45.2	
male	181	53.7	85	52.1	92	54.8	
Stage							
I	59	17.5	28	17.2	30	17.9	
II	137	40.7	61	37.4	73	43.5	
III	87	25.8	42	25.8	44	26.2	
IV	54	16.0	32	19.6	21	12.5	
Stage T							0.016
T1	7	2.1	5	3.1	2	1.2	
T2	59	17.5	26	16.0	32	19.0	
T3	235	69.7	106	65.0	124	73.8	
T4	36	10.7	26	16.0	10	6.0	
Stage M							0.105
M0	283	84.0	131	80.4	147	87.5	
M1	54	16.0	32	19.6	21	12.5	
Stage N							0.202
N0	203	60.2	92	56.4	107	63.7	
N1	76	22.6	37	22.7	38	22.6	
N2	58	17.2	34	20.9	23	13.7	

### Selection of m^5^C-Related Regulators

Based on published data, 14 m^5^C-related regulators, including NOP2 (NSUN1), NSUN2, NSUN3, NSUN4, NSUN5, NSUN6, NSUN7, DNMT2, TET1, TET2, TET3, ALKBH1, ALYREF, and YBX1 were used in our study. DNMT2 was not found to be expressed in COAD from TCGA datasets. Therefore, the remaining 13 m^5^C-related regulators were used for further analysis.

### Tumor Classification and Principal Component Analysis

To explore the function of m^5^C-related regulators in COAD, a consistent clustering algorithm was used to determine the clustering of samples and estimate the stability of the clustering. Using the “Consensus ClusterPlus” R package (Wilkerson and Hayes, 2010), two different subgroups (cluster Ⅰ and cluster Ⅱ) were identified based on the following classification parameters: 1) slow growth rate of the cumulative distribution function value; 2) high correlation in the subgroup; and 3) no small clusters in the clustering data. Furthermore, principal component analysis (PCA) was used to assess gene expression patterns in different subgroups using the “Limma” R package (Ritchie et al., 2015).

### Analysis of Clinicopathological Features and Prognosis

The correlation between m^5^C-related regulators and clinicopathological features was analyzed. Then, to filter the m^5^C-related regulators that were highly correlated with overall survival (OS), univariate Cox regression analysis was performed. Next, the Lasso Cox regression algorithm was used to identify m^5^C-related regulators with powerful prognostic significance. According to the best penalty parameter λ, the selected regulators’ coefficients were calculated. The risk score (RS) was estimated using the following formula:

$$RS=\sum_{i=1}^{n}Coef\left(i\right)X\left(i\right)$$

Where Coef(i) is the coefficient and X(i) represents the expression levels of the selected m^5^C-related regulators. Using the obtained median risk score as the demarcation value, patients with COAD were classified in two groups: high-risk group and low-risk group. The OS and clinicopathological features were compared between these subgroups. Kaplan-Meier analysis and the receiver operating characteristic (ROC) curves were used to validate the predictive efficiency (Hanley and McNeil, 1982). Additionally, the prognostic value of the RS was verified using univariate and multivariate Cox regression analyses. The hazard ratio (HR) with 95% confidence intervals and log-rank *p*-value were calculated using the “glmnet” and “survival” R packages (Simon et al., 2011).

### Biological Function Analysis

**FIGURE 1:**
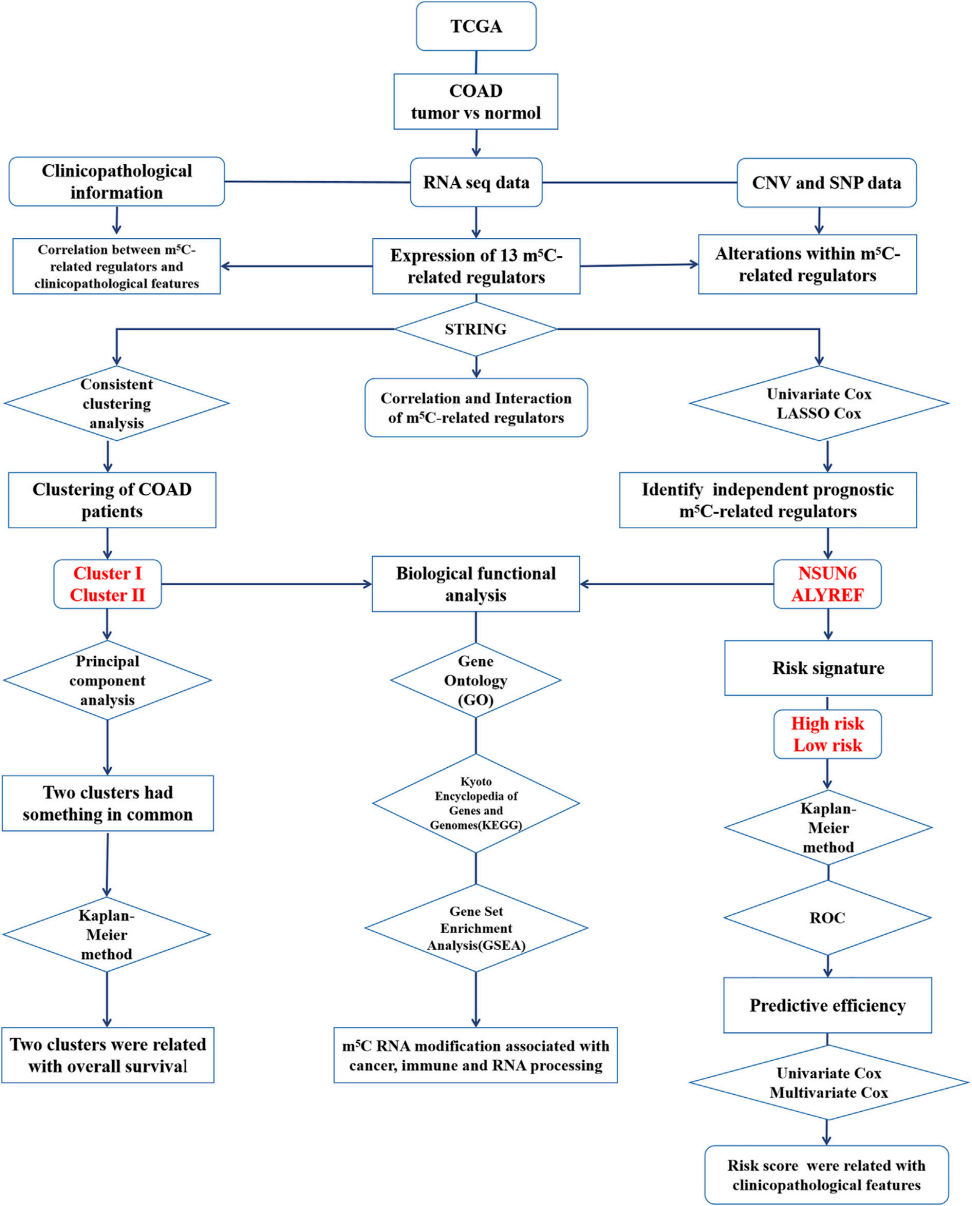
The flow chart of the study design and analysis.

To explore the biological functions associated with m^5^C RNA modification, Kyoto Encyclopedia of Genes and Genomes (KEGG) pathway analysis, Gene Ontology (GO) analysis and Gene Set Enrichment Analysis (GSEA) were performed. The genes that were differentially expressed between the high-risk group and the low-risk group were functionally annotated using GO analysis and KEGG pathway analysis. Next, GSEA was conducted to determine the signaling pathways related to different clusters. Later, to explore the latent biological function of the m^5^C-related genes in COAD, GSEA for the m^5^C-related regulatory genes with powerful prognostic value was performed. The flow chart of bioinformatic analysis was shown in Figure 1.

### Cell Culture

The COAD cell lines LS174T and normal colon mucosal epithelial cell line NCM460 were purchased from the American Type Culture Collection (ATCC, Manassas, VA, United States). All cells were cultured in RPMI-1640 medium (Life Technologies, Grand Island, NY, United States) supplemented with 10% fetal bovine serum (Life Technologies) at 37°C in a humidified atmosphere with 5% CO_2_.

### Quantitative Reverse Transcription-Polymerase Chain Reaction (qRT-PCR)

Total RNA was extracted from cells with TRIzol reagent (Invitrogen, China) according to manufacturer’s instruction. Reverse transcription was carried out according to the manufacturer’s instructions using the PrimeScript RT Reagent Kit (Takara, China). The SYBR PrimeScript RT-PCR Kit (Takara) was applied for the analysis of quantitative reverse transcription-polymerase chain reaction (qRT-PCR). Related mRNAs expression levels were calculated using the 2-ΔΔCT method and the related GAPDH mRNA expression was used as an endogenous control. Primers sequences used in our study were as follows: GAPDH forward 5′-GGA​CCT​GAC​CTG​CCG​TCT​AG-3′, and reverse 5′-GTA​GCC​CAG​GAT​GCC​CTT​GA-3′; NSUN6 forward 5′-TTT​GCC​ATC​TGC​CTT​AGT-3′, and reverse 5′-GTG​TGT​TGT​TTT​CCC​TCC-3′; ALYREF forward 5′-GCA​GGC​CAA​AAC​AAC​TTC​CC-3′, and reverse 5′-AGT​TCC​TGA​ATA​TCG​GCG​TCT-3′.

### Validation of the Protein Expression Levels of the m^5^C-Related Regulators *via* the Human Protein Atlas

To verify the protein expression levels of NSUN6 and ALYREF in COAD and normal tissues, immunohistochemistry (IHC) data were downloaded from the Human Protein Atlas (HPA, http://www.proteinatlas.org). The HPA online database provides IHC expression data for nearly 20 different cancers (Asplund et al., 2012) and enables the validation of the differential protein expression levels between tumor and normal tissues.

### Statistical Analysis

The expression data of m^5^C-related regulators in tumor tissues and adjacent mucosa of COAD obtained from TCGA were compared using one-way analysis of variance (ANOVA); the clinical characteristics and m^5^C-related regulators of different groups were compared using the chi-square test; the Kaplan-Meier method was used to perform a bilateral logarithmic rank test in overall survival analysis; *p*-values < 0.05 were regarded as statistically significant. All statistical analyses were implemented using Rv4.0.3 (https://www.r-project.org/).

## Results

### RNA-Seq Transcriptome Data of m^5^C-Related Regulators in COAD

**FIGURE 2:**
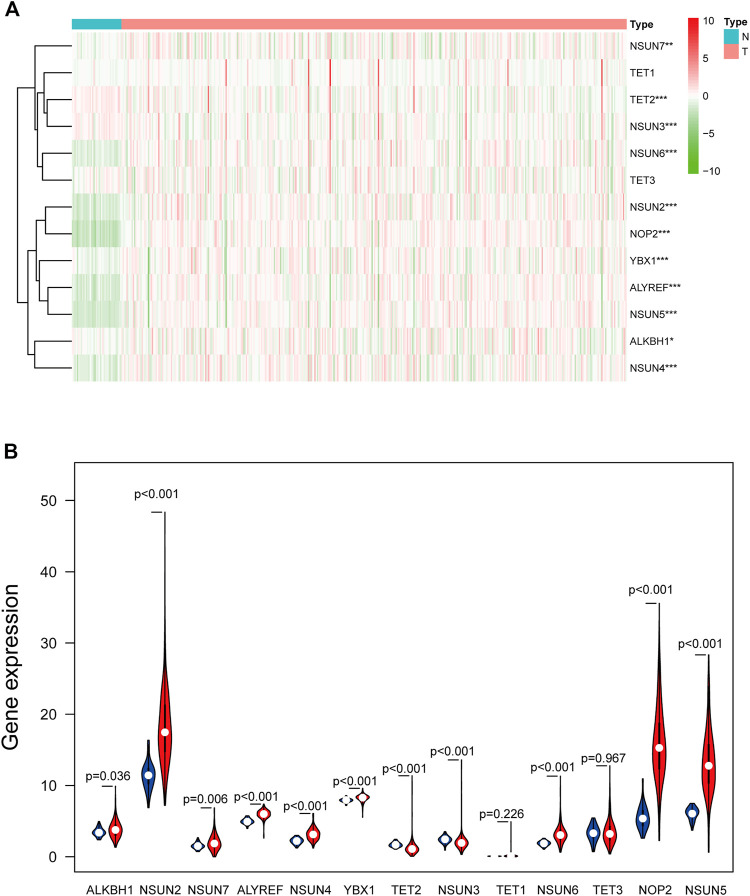
The expression of 13 m^5^C-related regulators in TCGA database between the tumor group and the normal group. **(A)** Heatmap of the expression of 13 m^5^C-related regulators. The depth of red represents the level of high expression, and the depth of green represents the level of low expression *p < 0.05, **p < 0.01, ***p < 0.001. **(B)** The violin diagram showed the median expression of 13 m^5^C-related regulators in COAD, and the position of white spots on the way represented the median value of the expression.

Based on RNA-seq transcriptome data of COAD from TCGA database, the expression of 13 m^5^C-related regulators between tumor tissues and adjacent mucosa was compared (Figure 2). With the exceptions of TET1 and TET3, the expression levels of the other 11 factors were significantly different in the tumor tissues and the adjacent mucosal tissues. Compared with the adjacent mucosa, the expression of NSUN3 (*p* < 0.001) and TET2 (*p* < 0.001) in the tumor group was significantly downregulated. The expression of ALKBH1 (*p* = 0.036), ALYREF (*p* < 0.001), NOP2 (*p* < 0.001), NSUN2 (*p* < 0.001), NSUN4 (*p* < 0.001), NSUN5 (*p* < 0.001), NSUN6 (*p* < 0.001), NSUN7 (*p* = 0.006), and YBX1(*p* < 0.001) were significantly upregulated in tumor tissues compared with the adjacent mucosa.

### Correlation and Interaction of m^5^C-Related Regulators in COAD

**FIGURE 3:**
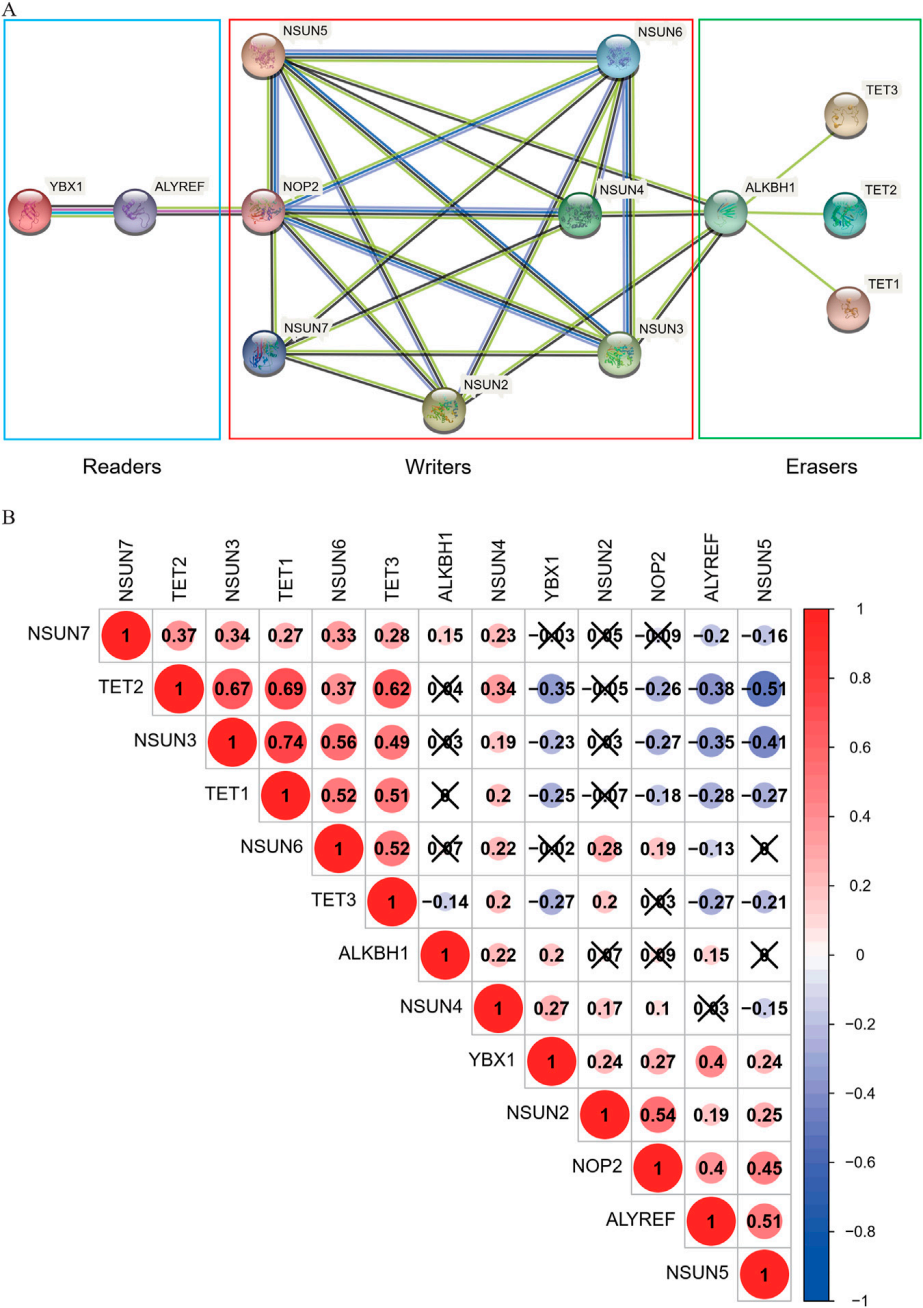
Correlation and interaction of m^5^C-related regulators in COAD. **(A)**The PPI network of the 13 m^5^C-related regulators was constructed using STRING. **(B)** Spearman correlation analysis of the 13 m^5^C-related regulators.

The correlations between the m^5^C-related regulators were analyzed using the “corrplot” package in R and their interrelationships were retrieved from the STRING database (https://string-db.org/). The expression levels of the seven “writers” were correlated with each other, except for NSUN2 and NSUN7, NSUN5 and NSUN7, NSUN2 and NSUN3, and NSUN5 and NSUN6. There were also close and complicated relationships between each regulator in the protein-protein interaction (PPI) network. We also found that the expression of TET family genes (TET1, TET2, TET3) were highly related to each other and had little correlation with ALKBH1. However, the TET family was associated with ALKBH1 in the PPI network and had interrelationships with the “writer” genes *via* ALKBH1. In addition, there was evidence supporting the interaction between the “reader” genes ALYREF and YBX1 in the PPI network. The expression of these genes was also positively associated with each other (Figure 3).

### CNVs and SNPs of m^5^C-Related Regulators in COAD

**FIGURE 4:**
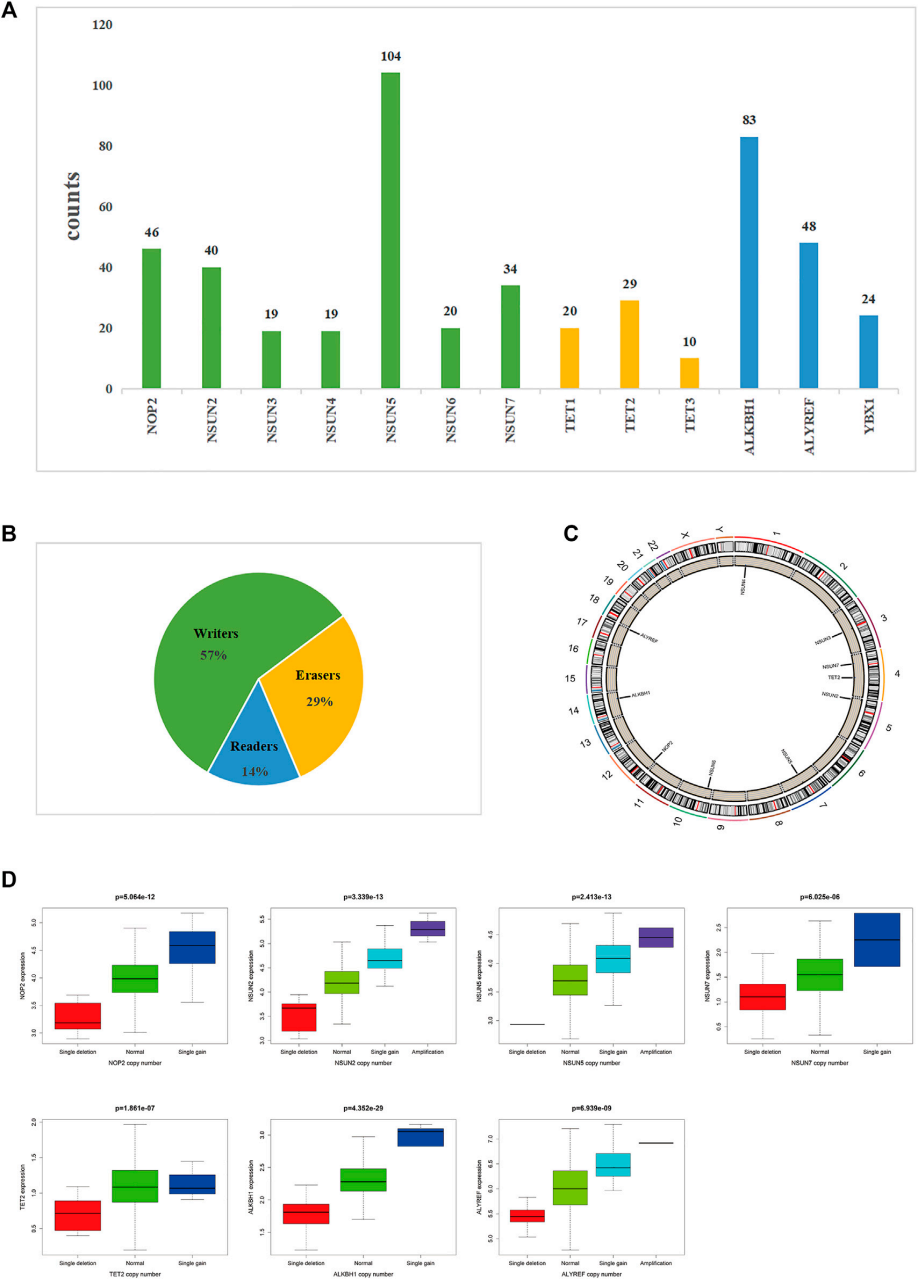
The landscape of CNV of m^5^C-related regulators in COAD.**(A,B)** Frequency of CNV of 13 m^5^C-related regulators in COAD. **(B)** Percentage of CNV of 13 m^5^C-related regulators in COAD. **(C)** Location of CNV alteration of 13 m^5^C-related regulators on chromosomes. **(D)** NOP2, NSUN2, NSUN5, NSUN7, TET2, ALKBH, and ALYREF displayed a significant difference in expression due to CNVs.

Regarding CNVs, we found that 10 of the 13 m^5^C-related regulators were significantly different between the tumor tissue and the adjacent mucosa from 825 samples with CNV data. Furthermore, it was found that CNVs affect the expression of m^5^C-related regulators. The highest frequency of CNVs occurred in the “writer” gene NSUN5 (24.47%), followed by the “eraser” gene ALKBH1 (19.53%). The “eraser” gene TET3 had the lowest CNV frequency (2.35%) (Table 2 **)**. The “writer” genes NOP2, NSUN2, NSUN5, and NSUN7, the “eraser” genes TET2 and ALKBH, and the “reader” gene ALYREF displayed a significant difference in expression due to CNVs (Figure 4).

**TABLE 2:** Copy number variants (CNV) of m5C related regulators in colon adenocarcinoma.

Function	Genes	Diploid	Deletion	Amplification	CNV sum	Deletion	Amplification	Percentage
Writers	NOP2	379	6	40	46	13.04%	86.96%	10.82%
	NSUN2	385	7	33	40	17.50%	82.50%	9.41%
	NSUN3	406	5	14	19	26.32%	73.68%	4.47%
	NSUN4	406	17	2	19	89.47%	10.53%	4.47%
	NSUN5	321	1	103	104	0.96%	99.04%	24.47%
	NSUN6	405	9	11	20	45.00%	55.00%	4.71%
	NSUN7	391	32	2	34	94.12%	5.88%	8.00%
Erasers	TET1	405	13	7	20	65.00%	35.00%	4.71%
	TET2	396	26	3	29	89.66%	10.34%	6.82%
	TET3	415	2	8	10	20.00%	80.00%	2.35%
	ALKBH1	342	78	5	83	93.98%	6.02%	19.53%
Readers	ALYREF	377	13	35	48	27.08%	72.92%	11.29%
	YBX1	401	19	5	24	79.17%	20.83%	5.65%

**FIGURE 5:**
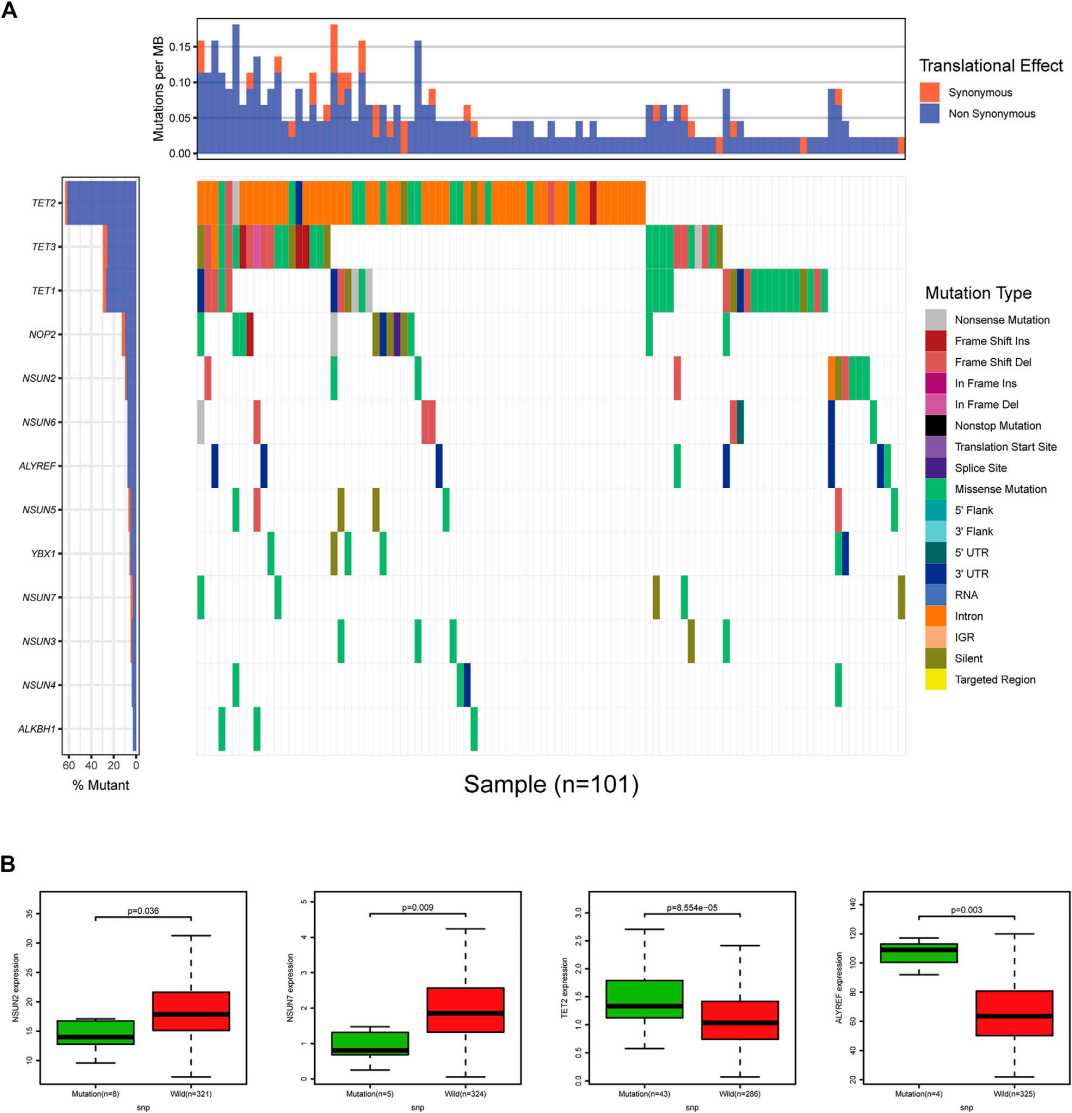
The landscape of SNP of m^5^C-related regulators in COAD. **(A)** Waterfall plot of SNP of 13 m^5^C-related regulators in COAD. **(B)** NSUN2, NSUN7, TET2, and ALYREF displayed significant differences in expression levels due to SNPs.

Regarding SNPs, we found that all of the m^5^C-related regulators had missense mutations, and missense mutations were the highest frequency mutation in 399 COAD cases with available sequencing data. Among them, the m^5^C “eraser” gene TET2 had the highest frequency of mutation events (96/399), followed by TET3 and TET1 (both 39/399). In addition, the “writer” genes NSUN2 and NSUN7, the “eraser” gene TET2, and the “reader” gene ALYREF displayed significant differences in expression levels due to SNPs. Next, we evaluated the effect of SNPs on patient prognosis, but no difference was observed due to the relatively few numbers of mutations (Figure 5).

### Consensus Clustering of Patients With COAD

**FIGURE 6:**
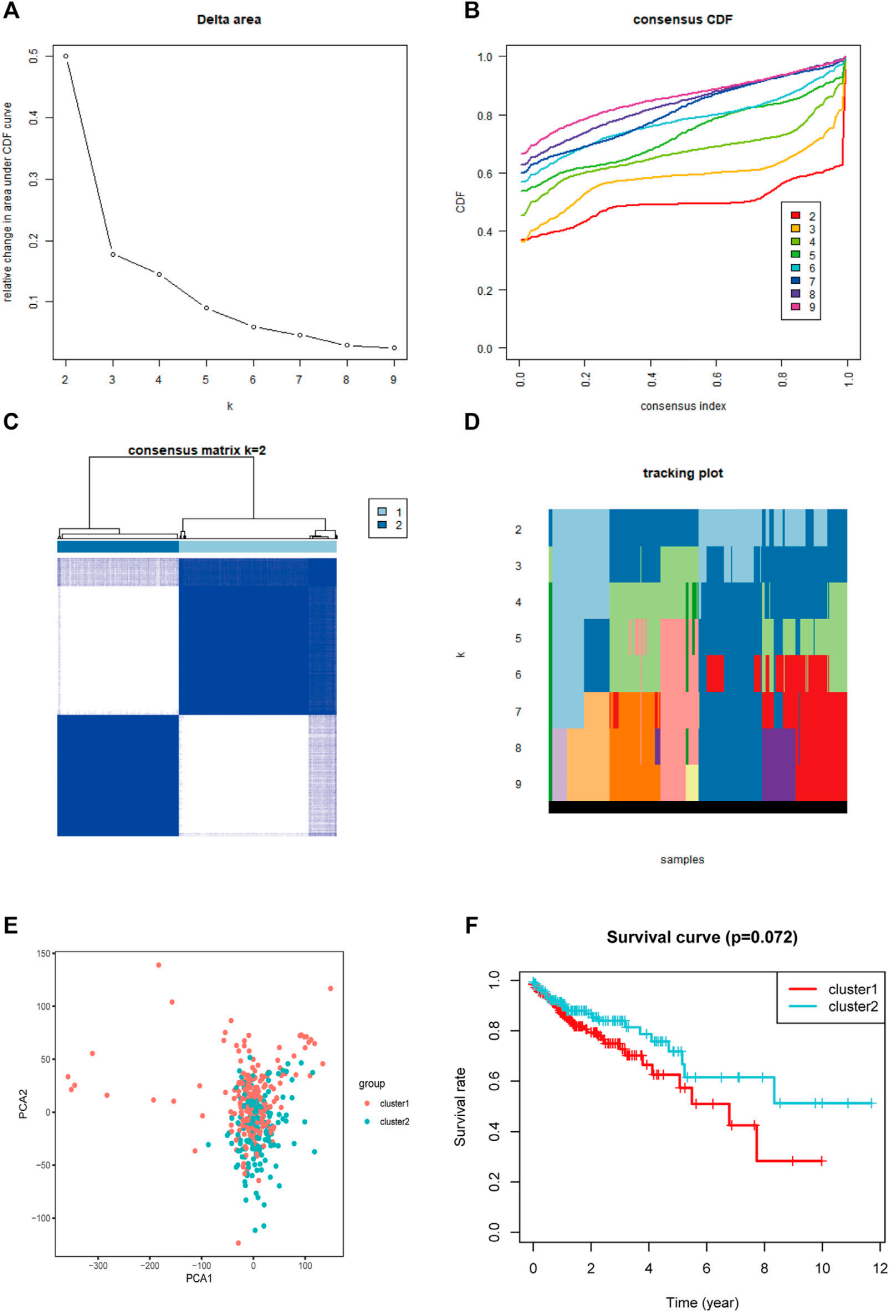
Consistent cluster analysis and principal component analysis of COAD. **(A)** The consistency clustering cumulative distribution function (CDF) when k is between 2 and 10. **(B)** The relative change of the area under the CDF curve from 2 to 10 of k. **(C)** At k = 2, the correlation between groups. **(D)** The distribution of the sample when k is between 2 and 10. **(E)** Principal component analysis of 2 clusters of total RNA expression profile after consistency analysis. **(F)** Comparison of Kaplan-Meier overall survival curves for COAD patients in cluster Ⅰ and Ⅱ.

Based on the expression levels of 13 m^5^C-related regulators, consistent clustering analysis of patients with COAD was performed, and they were clustered into two subgroups because there was minimal interference between the two subgroups (Figures 6A–D).

PCA showed that the RNA expression levels in patients with COAD in clusters I and II were specific (Figure 6E). Nevertheless, there were many overlapping areas between each cluster on the whole, indicating that the clusters had something in common. The cluster II had a longer survival time than cluster I when analyzed using the Kaplan-Meier method, but they had no significant different (Figure 6F).

### Prognostic Value of m^5^C-Related Regulators in COAD Prognosis

**FIGURE 7:**
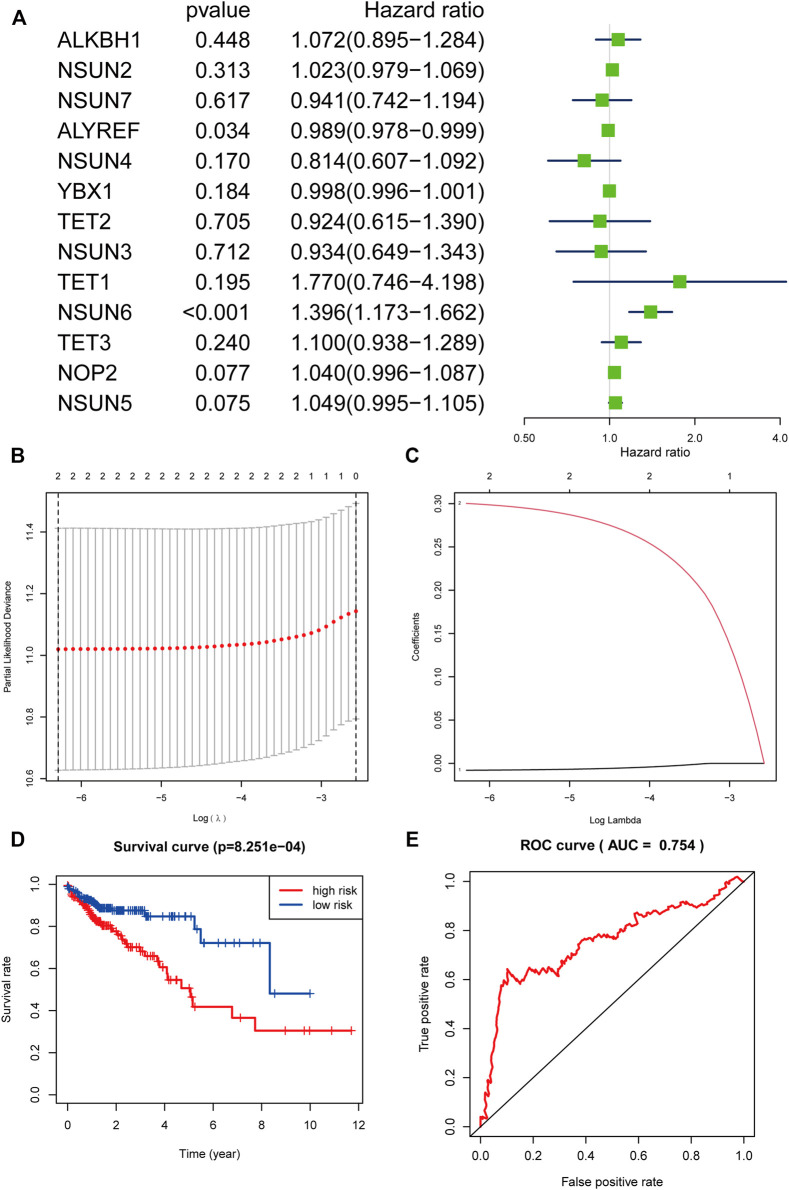
The process of constructing the signature based on NSUN6 and ALYREF and evaluating its prognostic value. **(A)** The Hazard ratio (HR), 95% confidence interval (CI) of 13 m^5^C-related regulators estimated by univariate Cox regression. **(B)** The point with the smallest cross verification error corresponds to the number of factors included in the Lasso regression model. **(C)** The lines of different colors represent the trajectory of the correlation coefficient of different factors in the model with the increase of Log Lamda. **(D)** Kaplan-Meier overall survival curves for patients in high-risk group- and low-risk group divided according to the risk score. **(E)** ROC analysis and AUC value of the ROC curve suggested the sensitivity and specificity for risk signature.

To evaluate the prognostic value of these 13 m^5^C-related regulators in COAD, univariate Cox regression analysis was used to identify m^5^C-related regulators that were highly correlated with the OS in patients with COAD, and two regulators with prognostic significance (*p* < 0.05) were found: NSUN6 and ALYREF. Specifically, ALYREF was considered a protective factor with HR < 1 in patients with COAD, and NSUN6 was considered as a risk factor with HR > 1 (Figure 7A). To further evaluate the prognostic significance of these two m^5^C-related regulators, LASSO Cox regression analysis was performed and it was revealed that NSUN6 (Coef = 0.300256795278519) and ALYREF (Coef = 0.00796895949684636) could serve as powerful prognostic factors in COAD (Figures 7B–C).

Based on NSUN6 and ALYREF, a risk signature was constructed and the risk score was calculated. Using the median risk score as the demarcation value, patients with COAD (*n* = 525) were classified into two groups, namely the high-risk and low-risk groups. To test the prognostic role of the two gene risk signatures, survival and ROC curve analyses were conducted. Based on the Kaplan-Meier (KM) survival analysis, the low-risk group had significantly longer survival time than the high-risk group (Figure 7D). In particular, compared with the 46.4% 5-year survival rate in the high-risk group, that of the low-risk group was 78.7%. The area under the curve (AUC) value in the time-dependent ROC curve was 0.754, suggesting good prediction performance of the survival model (Figure 7E).

### Correlation Between the Two m^5^C-Related Regulators, Risk Score, and Clinicopathological Characteristics in COAD

**FIGURE 8:**
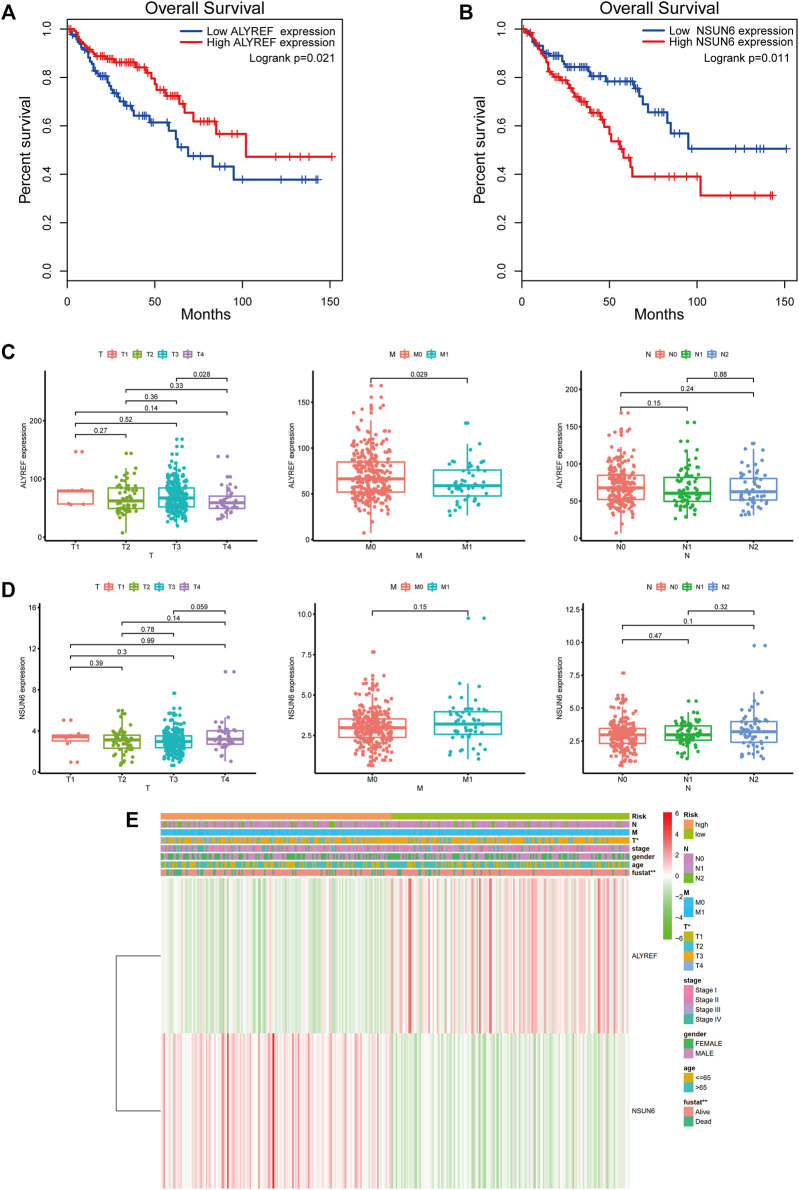
Survival analysis and clinicopathological characteristics of the two m^5^C-related regulators. **(A)** Kaplan-Meier survival curve of ALYREF in high- and low-expression groups. **(B)** Kaplan-Meier survival curve of NSUN6 in high- and low-expression groups. **(C)** Analysis of the relationship between the expression of ALYREF and TMN stage. **(D)** Analysis of the relationship between the expression of NSUN6 and TMN stage. **(E)** The heatmap shows the expression of NSUN6 and ALYRE in high-risk and low-risk. The distribution of clinicopathological characteristics was compared between the high-risk and low-risk groups. *p < 0.05, **p < 0.01.

We further analyzed the relationship between the two m^5^C-related regulators, risk score, and different clinical variables. KM survival analysis showed a close association of the two m^5^C-related regulators (NSUN6 and ALYREF) with the OS of patients with COAD (Figures 8A,B). In terms of TMN stage, the expression of ALYREF was differentially expressed between T3 stage and T4 stage and between M0 stage and M1 stage (Figure 8C). However, the expression of NSUN6 was not significantly different across groups in the TMN stage (Figure 8D). The expression of the two m5C-related regulators and the distribution of clinicopathological characteristics in the high-risk and low-risk groups are displayed as a heatmap (Figure 8E). Evident differences between the two groups according to stage T (*p* < 0.05) and fustat (*p* < 0.01) were observed.

**FIGURE 9:**
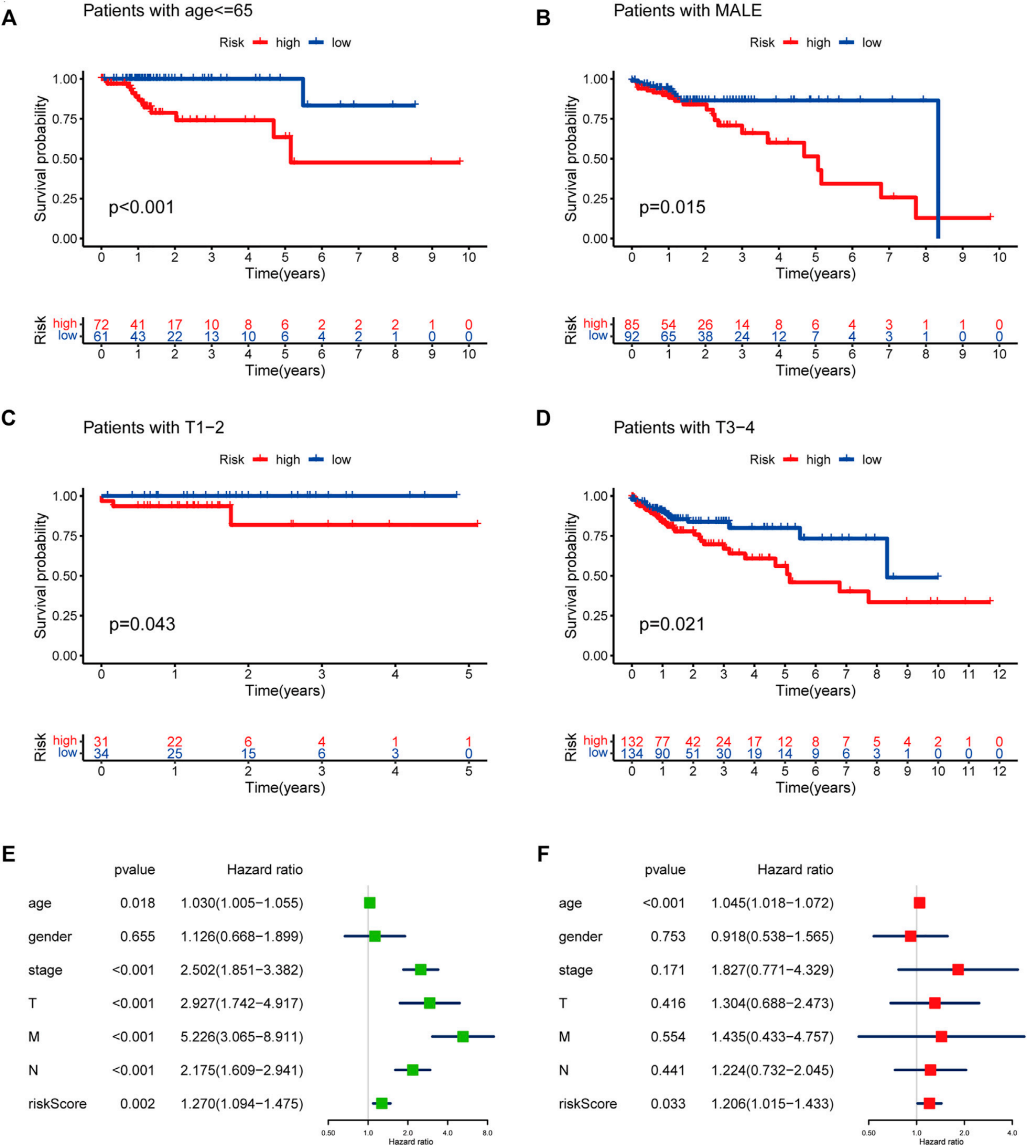
Subgroup analysis with risk score in different clinicopathological features and Prognostic risk model verification. **(A)** age ≤ 65. **(B)** male. **(C)** T1-T2. **(D)** T3-T4. **(E)** Univariate Cox regression analysis of risk score combined with clinicopathological factors. **(F)** Multivariate Cox regression analysis of risk score combined with clinicopathological factors.

To evaluate whether the risk score could serve as a prognostic indicator for OS in subgroups of patients with different clinical characteristics, we stratified subgroups by age (age ≤ 65 and age > 65), gender (female and male), clinical stage (stage I-II and stage III-IV), stage T (T1-2 and T3-4), stage M (M0 and M1) and stage N (N0 and N1-2). As the result shown in Figures 9A–D, the OS of the low-risk patients based on age (*p* < 0.001 in age ≤ 65), sex (*p* < 0.001 in male), and stage T (*p* < 0.005 in stage T1-2 and T3-4) was significantly higher than those of the high-risk patients.

To further examine whether the risk score was an independent prognostic factor, univariate and multivariate Cox regression analyses were conducted. This revealed that the risk score was significantly associated with OS in univariate analysis, in addition to age at diagnosis, pathological stage, and TNM stage (*p* < 0.05). However, only the age at diagnosis and risk score were correlated with OS (*p* < 0.05) in the multivariate Cox regression analysis (Figures 9E,F).

### Biological Functional Analysis

As we clustered the patients with COAD into cluster Ⅰ and cluster Ⅱ, genes that were significantly upregulated (fold change >1 and *p* < 0.05) or downregulated (fold change <1 and *p* < 0.05) between the high-risk group and low-risk group were identified using the “edgeR” package in R. GO and KEGG pathway analysis were used for biological functional analysis.

**FIGURE 10:**
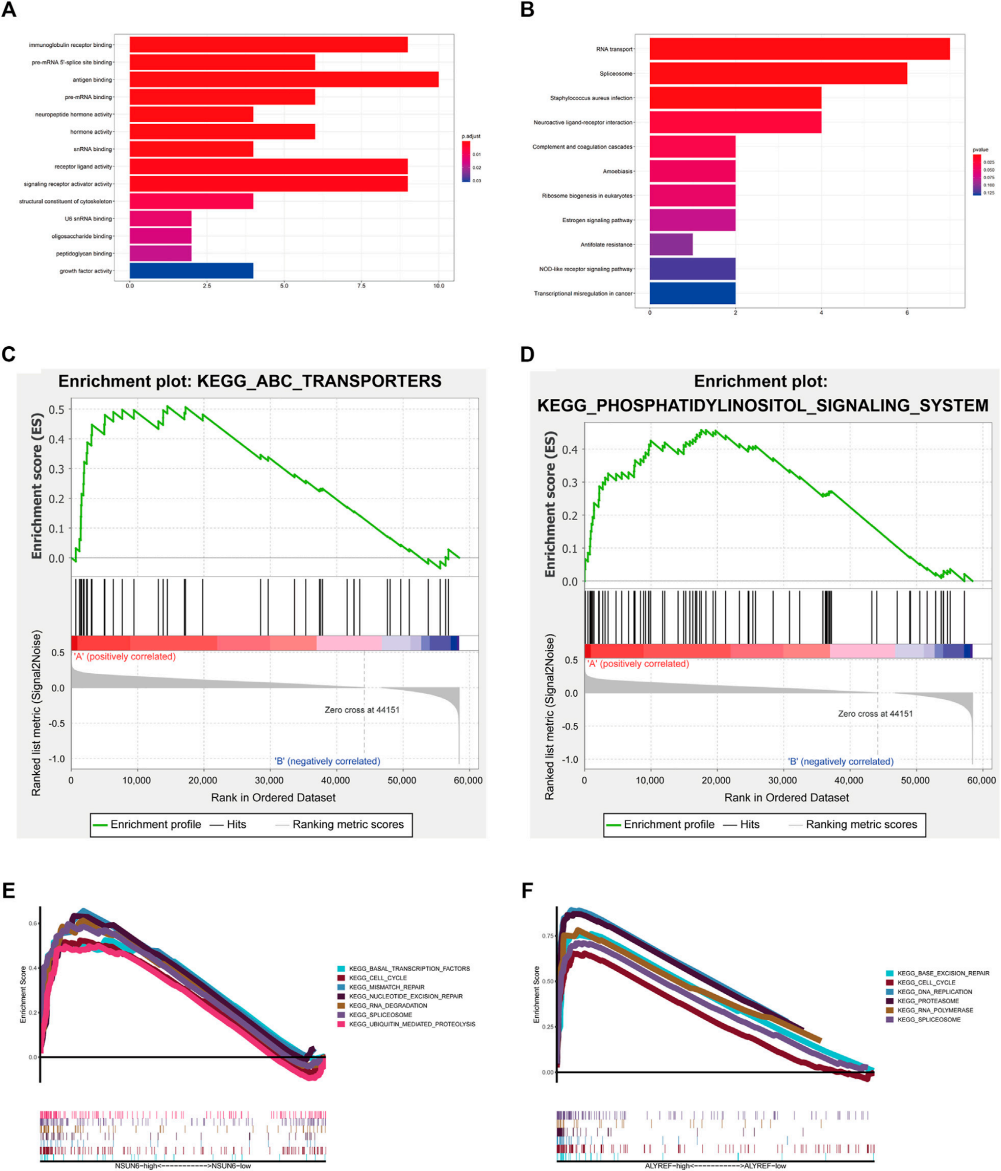
Biological functional analysis. **(A,B)** GO analysis and KEEG pathways analysis of the genes significantly upregulated or downregulated between cluster Ⅰ and cluster Ⅱ. **(C,D)** Cluster I had a worse overall survival and lower 5-year survival rate associated with malignancy-associated pathways, including the ATP-binding cassette transporter and phosphatidylinositol signaling system. **(E)** GSEA results for NSUN6 in COAD. **(F)** GSEA results for ALYREF in COAD.

Concerning GO analysis, the differentially expressed genes were associated with immune-related biological processes, such as “antigen binding” and “immunoglobulin receptor binding,” and pre-mRNA-related biological processes, such as “pre-mRNA 5′-splice site binding” and “pre-mRNA binding.” (Figure 10A). KEGG pathway analysis results were correlated with immune-related pathways, including “complement and coagulation cascades” and “NOD-like receptor signaling pathway,” and RNA-related pathways, including “RNA transport” and “spliceosome.” Moreover, cancer-related pathways were enriched, such as “transcriptional misregulation in cancer” and “MAPK signaling pathway” (Figure 10B).

Next, we used GSEA to predict the functional difference between clusters I and II. The results showed that cluster I had a worse OS and lower 5-year survival rate associated with malignancy-associated pathways, including the ATP-binding cassette transporter (NES = 1.79, normalized *p* = 0.006) and phosphatidylinositol signaling system (NES = 1.63, normalized *p* = 0.03) (Figures 10C,D).

Furthermore, as NSUN6 and ALYREF were shown to be important regulators of m^5^C in our study, GSEA was performed to investigate the potential biological processes associated with NSUN6 and ALYREF in COAD pathogenesis. GSEA suggested that increased expression of NSUN6 and ALYREF is involved in various biological functions in RNA processing, such as spliceosome, RNA polymerase, and RNA degradation. Upregulation of these genes was associated with malignancy-associated pathways, such as the cell cycle (Figures 10E,F).

### Validation of the Expression Levels of the m^5^C-Related Regulators in Cell Lines and Clinical Samples

**FIGURE 11:**
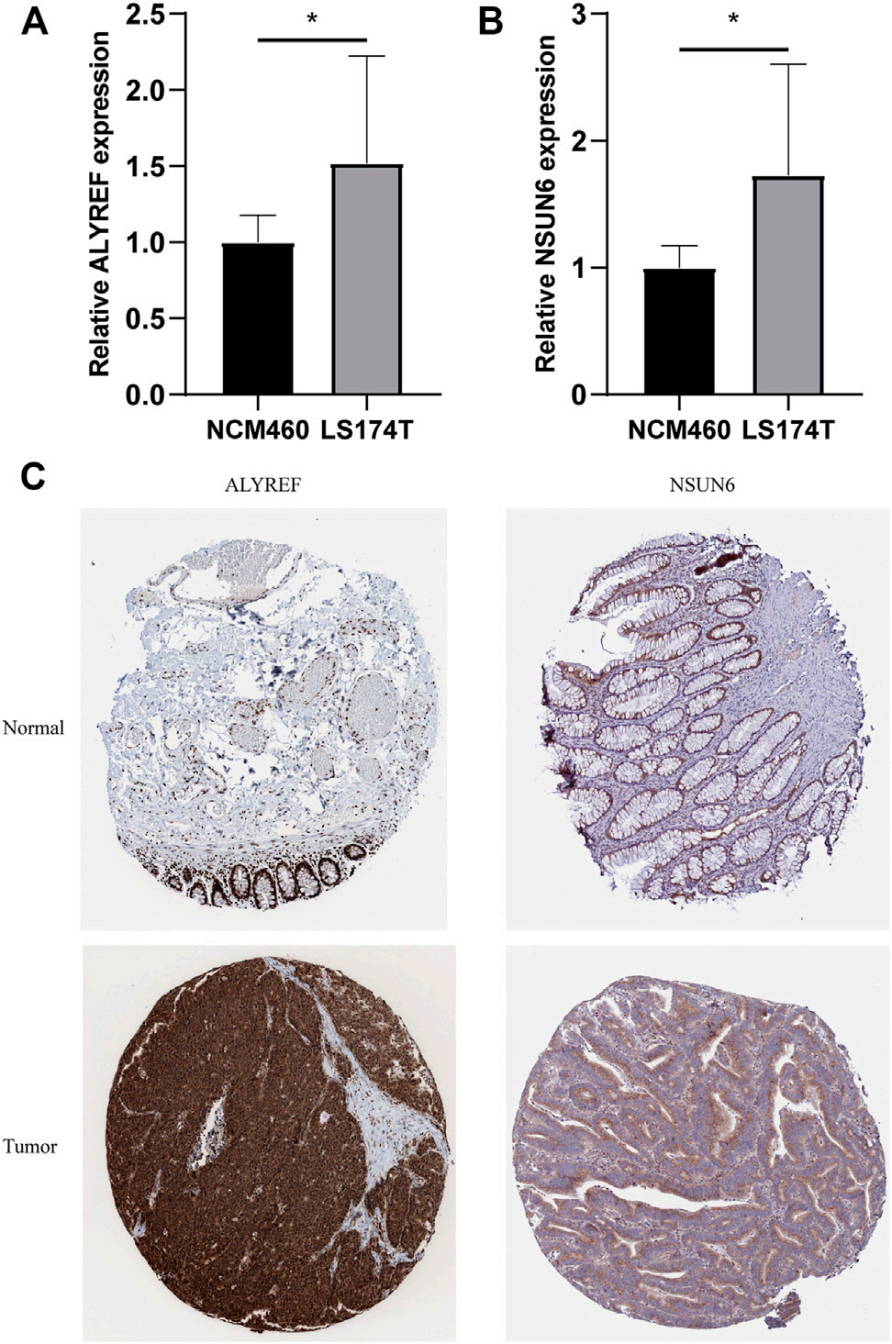
Validation of the Expression Levels of the m^5^C-Related Regulators in Cell Lines and Clinical Samples. **(A,B)** Expression of ALYREF and NSUN6 in COAD cell lines LS174T and normal colon mucosal epithelial cell line NCM460. *p < 0.01. **(C)** IHC analysis of the protein level expression of ALYREF and NSUN6 in COAD and normal tissues in HPA online database.

For validating the expression levels of the two m^5^C-related prognostic regulators from prognostic signature, we detected the expression levels in the COAD cell lines LS174T and normal colon mucosal epithelial cell line NCM460 by qRT-PCR. Our results showed that NSUN6 and ALYREF were significantly upregulated in LS174T compared with NCM460 (Figures 11A,B). IHC data from the HPA online database also demonstrated that the protein levels of NSUN6 and ALYREF were more highly expressed in cancer tissues than in normal tissues (Figure 11C).

## Discussion

RNA modifications have been increasingly demonstrated in tumorigenesis and tumor progression, suggesting that RNA epigenetic regulators may play an important role in COAD. Previous studies have shown that m^6^A RNA modification not only plays a critical role in the tumorigenesis and progression of CRC, but also has powerful significance in the diagnosis and prognosis of CRC patients (Li et al., 2021). Additionally, a growing body of evidence shows that m^5^C-related regulators could be latent predictive biomarkers in a variety of cancer (Huang et al., 2021a; Huang et al., 2021b; Pan et al., 2021). However, the literature on CRC and m^5^C has largely focused on DNA methylation (Zhu et al., 2018). Little is known about the relationship between m^5^C-related RNA modifications and CRC, which calls our attention to investigate the aberrant expression of m^5^C-related regulators in COAD and explore whether m^5^C-related regulators could serve as ideal biomarkers for COAD prognosis and participate in COAD initiation and progression.

In our study, we showed that the expressions of m^5^C-related regulators were significantly altered between tumor tissues and adjacent mucosa and had a strong correlation with the tumor progression and prognosis. This indicated that m^5^C-related regulators play a crucial role in COAD. First, the “writer” genes NSUN1-NSUN7, the “eraser” genes TET2 and ALKBH1, and the “reader” genes ALYREF and YBX1 were significantly upregulated or downregulated in tumor tissues, suggesting these genes may be critical in m^5^C-related occurrence and progression of COAD. To investigate the relationship between CNVs or SNPs of m^5^C-related regulators and their mRNA expression levels, COAD samples with CNV or SNP data from TCGA were analyzed. Regarding CNVs, the copy number of seven m^5^C-related regulators increased or was lost, and their mRNA expression was upregulated or downregulated accordingly and was significantly correlated. SNPs in TET2 and ALYREF were highly correlated with their high mRNA expression, while SNPs of NSUN2 and NSUN7 were significantly correlated with their low mRNA expression levels. Additionally, m^5^C-associated mutations in COAD could be studied in RMVar and RMdisease database, which were recently constructed and focused on genetic variants in RNA modifications (Kunqi Chenet al., 2021; Xin Luo et al., 2021).

Thereafter, based on the expression of the m^5^C-related regulators, patients with COAD were clustered into two subgroups (cluster Ⅰ and cluster Ⅱ), an the cluster II had a longer survival time than cluster I. To further study the effect of m^5^C-related regulators on the prognosis and clinicopathological characteristics of COAD, we constructed a prognostic risk signature using two identified m^5^C-related regulators (NSUN6 and ALYREF) and were able to assign patients with COAD into high- and low-risk groups. The correlation between the groups and clinicopathological characteristics was assessed, which revealed that the high-risk group was linked with stage T and fustat. Based on the risk value, the established ROC curve showed a satisfactory prediction performance. Moreover, the risk score can be used as an independent prognostic factor for COAD, suggesting that NSUN6 and ALYREF may be vital m^5^C-related regulators and significant prognostic factors for patients with COAD. Furthermore, this m5C-related regulators prognostic model could serve as a prognostic indicator for OS in subgroups of patients with different clinical characteristics, especially age ≤65, male, and stage T. The results presented above indicated that NSUN6 and ALYREF can be used as potential biomarkers, and a reliable risk model is critical for providing the necessary evidence for clinical adoption. Apart from our results, there was another study similarly demonstrated that a risk score developed from the three-m^5^C signature represented an independent prognostic factor for patients with COAD (Geng et al., 2021).

Recently, many studies have indicated that m^5^C RNA modification is involved in all types of human cancer. NSUN2 is the most studied m^5^C methyltransferase and participates in various cancers, such as bladder cancer, gallbladder carcinoma, and hepatocellular carcinoma (Chen et al., 2019; Gao et al., 2019; Sun et al., 2020). It was reported that NSUN2 is highly expressed in colon cancers (Okamoto et al., 2012), which was corroborated in our results. NSUN2 mainly exerts an oncogenic role by maintaining the stability of oncogenic RNA (Chellamuthu and Gray, 2020), but whether NSUN2 plays the same role in COAD requires further research. With respect to the two m^5^C-related regulators (NSUN6 and ALYREF) identified in our results, there have been some studies on cancer and related mechanisms. The role of NSUN6 in regulating cell proliferation and pancreatic cancer tumor growth was recently confirmed, and NSUN6 performs well in evaluating tumor recurrence and survival among pancreatic cancer patients (Yang et al., 2021). Next, ALYREF was found to be upregulated in hepatocellular carcinoma and oral squamous cell carcinoma, and it may have an effect on tumorigenesis *via* cell cycle regulation and mitosis (Saito et al., 2013; He et al., 2020).

To provide a comprehensive analysis, GO, KEEG pathway, and GSEA analyses of m^5^C-related regulators were also conducted. Several biological processes and pathways associated with the occurrence and progression of COAD were enriched, including “MAPK signaling pathway” and “cell cycle” (Koveitypour et al., 2019; Malki et al., 2020). Moreover, previous studies have reported that m^5^C-related RNA modifications are closely associated with mRNA translation, transport, and stability. Here, we found that the m^5^C-related regulators were associated with “pre-mRNA 5′-splice site binding” and “spliceosome,” suggesting they play important roles in RNA processing. In addition, it should be noted that a number of biological processes and pathways associated with immune response were identified. While extensive literature reports have demonstrated that N6-methyladenosine plays important role in immune evasion and immune response (Xiaoting Lou et al., 2021; Shulman and Stern-Ginossar, 2020) and bioinformatic analysis have shown that the m^5^C-related regulators were related to tumor immune microenvironment and affected the abundance of tumor-infiltrating immune cells in COAD (Geng et al., 2021), there have been few experimental reports about the relationship between m^5^C-related RNA modifications and immune response, suggesting that further research is required.

However, there are some limitations associated with our research. Firstly, the m^5^C-related regulators we selected included some DNA demethylase, such as TET1, TET2, TET3, and ALKBH1. The specific role of these genes in m^5^C RNA modification and DNA methylation of COAD and the crosstalk between m^5^C RNA modification and DNA methylation in COAD need to be further explored. Secondly, our research mainly focused on bioinformatic analysis, more experimental studies exploring the function of m^5^C on the different types of RNA and sites in COAD are in urgent need in future work. The m^5^C-Atlas database, a comprehensive database for decoding and annotating the m^5^C epitranscriptome, may be useful in the research (Ma et al., 2022). Thirdly, overall survival between cluster Ⅰ and cluster Ⅱ had no significant difference, more m^5^C-related regulators and more cohort need to be included in future analysis.

## Conclusion

In this study, we first found that there was a significant correlation between the expression of m^5^C-related regulators and clinicopathological features and OS of patients with COAD. This revealed that a prognostic signature obtained using m^5^C-related regulators (NSUN6 and ALYREF) had significant value in COAD and could effectively predict the survival of patients with COAD. Additionally, biological processes and pathways associated with m^5^C-related RNA modifications were identified, which may facilitate the malignant development of COAD, thus improving our understanding of the role of m^5^C-related RNA modifications in the occurrence and progression of COAD. This work also provides important evidence towards the development of predictive biomarkers and molecular targeted therapy for COAD Bray et al., 2018.

## Data Availability

Publicly available datasets were analyzed in this study, which can be found in the Cancer Genome Atlas (TCGA) database and the Human Protein Atlas (HPA) online database.
